# Transforming the Emotional Intelligence of the Feeders in Agribusinesses into the Development of Task Performance and Counterproductive Work Behaviors during the COVID-19 Pandemic

**DOI:** 10.3390/ani11113124

**Published:** 2021-10-31

**Authors:** Stanley Y. B. Huang, Ming-Way Li, Yue-Shi Lee

**Affiliations:** 1 Master Program of Financial Technology, School of Financial Technology, Ming Chuan University, Taipei 111, Taiwan; yanbin@mail.mcu.edu.tw; 2 Department of Marketing and Logistics Management, College of Business Management, Chihlee University of Technology, New Taipei 220, Taiwan; 3 Department of Computer Science and Information Engineering, Ming Chuan University, Taoyuan 333, Taiwan; leeys@mail.mcu.edu.tw

**Keywords:** counterproductive work behavior, emotional intelligence, task performance, transformational leadership, work burnout, work engagement

## Abstract

**Simple Summary:**

This research proposes a psychological model to describe how leadership can deal with the work burnout of feeders in livestock production agribusinesses to solve the important problems of mental health and well-being, thereby increasing the sustainable work of feeders. The empirical evidence comes from 240 livestock feeders from 80 Taiwanese livestock production agribusinesses. The research results can push the literature of emotional intelligence and implementation methods to livestock production agribusinesses.

**Abstract:**

The present research poses a novel multilevel model to describe how transformational leadership can significantly affect task performance and counterproductive work behavior through intermediary effects of emotional intelligence, work engagement, and work burnout. The empirical data is from 240 livestock feeders from 80 Taiwanese livestock production agribusinesses. The empirical results demonstrate that leadership could indeed transform the emotional intelligence of livestock feeders into positive task performance and negative counterproductive work behavior. The research results can provide an implementation method for livestock production agribusinesses to achieve the sustainable work of feeders in agribusinesses through handling task performance and counterproductive work behavior of feeders.

## 1. Introduction

### 1.1. Background

In the agricultural sector, livestock production has accounted for 33% of the gross production and 60% of the labor force in the world [1,2]. However, the sudden COVID-19 pandemic caused many agricultural production restrictions to avoid the outbreak of COVID-19, which also caused major disruptions in the agricultural production supply chain [3]. Therefore, it has caused difficulties in the production and sales of agricultural products and further caused the production workers in this field (such as the feeders of livestock production agribusinesses) to face huge unemployment pressure, which is the first source of work stress for feeders in agribusinesses. Indeed, Taiwan’s unemployment rate has reached a new peak in 11 years (4.8%) due to COVID-19 [4]. In addition, previous studies have examined that COVID-19 may infect the farm employees from livestock [5,6,7], so these livestock feeders need to face daily infections from livestock, which is the second source of work stress for feeders in agribusinesses. Table 1 demonstrates these variable acronyms in the present survey. 

Indeed, to obtain a superior competitive advantage, livestock production agribusinesses vendors must motivate their livestock feeders to use their full energy for performance (e.g., TP) and should also reduce their negative behaviors (e.g., WB) due to work pressure at the same time [8] to achieve the goal of sustainable work. Previous empirical studies have investigated the organizational enhance variables that increase positive behaviors [9,10,11] and organizational intervention variables that reduce negative employee behaviors [12,13,14]. However, few studies focus on how a single variable (e.g., EI) can simultaneously handle the positive and negative behaviors of feeders in agribusiness. In addition, although past research has paid attention to EI in the multidisciplinary field [15,16,17], EI has almost been seen as a leading variable for predicting positive behavior in previous studies [18,19,20]. EI indicates the capability of individuals to manage their emotion-related behaviors [15,17]. However, few studies have explored TL as the antecedent of EI [21]. TL indicates that a leader uses ideal infection, inspiration, individual concern, and intelligent excitation to transform employees for resulting high-level thinking and performance [22]. The process of TL should include EI, and this assumption is also supported by social learning theory [23]. For example, these feeders in agribusinesses may passively imitate EI behavior from their supervisors through the TL process to increase their EI, which has also been examined in previous research [24].

Researchers in the past have been lacking in exploring the key antecedents that can increase employees’ WE and TP and can improve employees’ WB and CWB at the same time. WB indicates a psychological state of spirit exhaustion [25]. CWB indicates that employees use negative behaviors to vent their uneasy emotions to harm the company or colleagues [26]. WE is a job-related mental condition in which individuals put their selves into the job [27]. However, few studies have studied both intervention strategies in mitigating the development of WB and enhancement strategies in promoting the development of WE at the same time, leading to key literature gaps. Indeed, WE and WB are not just trivial concepts because these two concepts will affect performance and turnover intention, which will cause significant losses to the company [28,29,30]. Past researchers mainly employ job-person fit and work demand-resource perspectives as intervention strategies to alleviate WB [31,32] and employ the work demand-resource views and Kahn’s [27] framework as enhancement strategies to increase WE [33,34,35]. In response to this list, the second target of this research is to propose a new stream that uses EI to simultaneously alleviate WB and enhance WE, thereby affecting CWB development and TP development. Indeed, it is important to study how EI simultaneously increases WE and reduces WB because it can not only increase outstanding performance but also achieve the goal of sustainable work of feeders in agribusinesses to realize the competitive advantage. In addition, previous investigations focused less on the impact of TL, EI, WE, and WB at the organizational level to individual-level CWB and TP, so the present research extends these variables to the work-unit level to open the black box of psychological mechanisms more completely. Therefore, this research proposes the multilevel model [36,37,38], which surveys how cross-level TL can significantly affect cross-level EI, WE, and WB, thereby leading to CWB and TP. Indeed, past surveys have empirically explored the antecedents of TP and CWB [39,40,41], but few studies pay attention to the antecedents of CWB and TP at the work-unit level. The present research surveyed 240 livestock feeders from 80 Taiwan livestock production agribusinesses and analyzed the proposed model through the multilevel model to fill this concern. In particular, EI, WE, TP, WB, and CWB were seen as individual-level variables in the past studies [42,43,44,45,46,47,48,49,50,51] and cross-sectional samples [52,53,54]. By using the multilevel model to analyze the data, this research can handle the literature gaps in social science research. 

### 1.2. Research Framework and Development of Hypotheses

#### 1.2.1. TL and EI

Past researchers [22] found that TL has four dimensions, including ideal infection, inspiration, individual concern, and intelligent excitation, to transform employees. Previous studies have shown that TL can increase positive behaviors of employees [55,56,57] because TL can change employees’ self-worth to meet organizational expectations. However, past research has paid little attention to the effect of the TL process for EI because TL can change an employee’s self-worth and capability by adjusting and expressing appropriate emotions (i.e., EI) in a work setting to attain good performance. That is to say, a leader uses ideal infection, inspiration, consideration, and intelligent excitation to transform an employee’s self-worth to meet organizational expectations, and it can strengthen the employee’s EI capability, which regulates and shows appropriate emotions. In addition, a transformational leader may express appropriate emotions to express concern, and subordinates may imitate such emotions. Indeed, social learning theory also supports this hypothesis [23] because an employee may passively learn behaviors from his colleagues or supervisor to obtain a reward instead of a punishment. Past research has also found that an individual can learn EI behaviors from others [58] and TL may influence EI [59].

**Hypothesis** **1.** *TL will affect EI.*

#### 1.2.2. EI, WE, and WB

WE means a psychological state of fulfillment, both positive and work-related, in which an individual can give his or her full self in achieving role performance of job through putting physical, cognitive, and emotional resources into the work [27]. When a person is physically, cognitively, and emotionally attached to the work, he or she will show WE [27]. A person with a high WE value is energetic and has a high sense of self-efficacy because he or she has a proactive attitude towards work. One possible reason for the positive impact of EI on WE is that EI is a psychological resilience or personal resource [60] and a person experiences long-term positive emotions that can increase his or her mental resilience [61,62]. Indeed, positive emotions can expand his or her initial thoughts and possible actions to cope with good or bad situations. Broadly speaking, EI is the capability to know the emotional needs of others and how to cope with stress by regulating and expressing emotions that can cope with multiple situations [63]. Personal resources can stimulate the motivational process that can affect willingness to work [64], thereby increasing the individual’s willingness to devote himself or herself to his or her role performance (i.e., WE). Indeed, a previous study also assumed that personal resources have a positive impact on WE [65].

**Hypothesis** **2.** *EI will affect WE.*

WB includes depersonalization (personal attitude is to keep a distance from the stressor) and emotional exhaustion (a mental state of exhaustion) [25,66,67]. A person who has experienced WB may show negative emotions, lack of energy, and low motivation for work and customers [25] and believe that his performance has declined.

Previous empirical research has proposed that the correlation between EI and WB is significantly negative [68], and has also proposed that EI may reduce WB [69]. In addition, based on the resource perspective discussed earlier, some research evidence also suggests that resources may have a negative impact on WB [31]. Indeed, insufficient resources may lead an individual to experience the status of WB [70]. In contrast, adequate resources may cause individuals to avoid resource depletion [71]. On the other hand, insufficient resources will lead to difficulties in obtaining resources, leading to WB.

**Hypothesis** **3.** *EI* *will* *affect WB.*

#### 1.2.3. WE and TP

TP means an employee’s activities to complete core tasks [72]. A dedicated employee should devote more resources to his or her work than a less dedicated employee, and the dedicated employee should generate more performance across fields, such as working with external experts or searching for external resources. Indeed, WE means focus, vitality, and dedication, and an employee with high WE levels must cultivate complete and positive performance through innovation, interpersonal cooperation, and participation in work [73].

**Hypothesis** **4.** *WE will affect TP.*

#### 1.2.4. WB and CWB

CWB is an employee’s relief of anxiety through negative behaviors that may harm the company or colleagues [26]. Although few studies have explored how WB affects CWB, several studies provide some possible evidence for this relationship. Past studies have found that emotionally exhausted employees often exhibit CWB [74] because CWB is a side effect of WB. In addition, Krischer and his colleague [75] found that employee CWB may be a way to vent the emotional exhaustion of employees because employees who deal with abnormal production behaviors show less emotional exhaustion than employees who avoid abnormal production behaviors. However, Krischer and his colleague [75] did not test the statistical significance between WB and CWB. In addition, the theory of conservation resources [76] believes that there is a connection between WB and CWB. Because an emotionally exhausted employee (i.e., high levels of WB) lacks resources, the employee may save his or her resources by rejecting production behavior to show CWB.

**Hypothesis** **5.** *WB will affect CWB.*

#### 1.2.5. Multilevel TL, EI, WE, and WB

TL, EI, WE, and WB can be examined for the multilevel from a theoretical and empirical perspective. Indeed, previous surveys have investigated TL, EI, WE, and WB as multilevel [77,78,79,80] by compiling the individual-level employee perception of these variables based on the multilevel method perspective [81]. Indeed, TL, EI, WE, and WB should be investigated at the work-unit level because these variables may appear in specific environments that cannot be investigated only at the individual level [82]. In addition, socialization theory also supports this hypothesis that new members of the working group can learn from other members through interaction to cultivate homogenous perception [83], thereby supporting the TL, EI, WE, and WB at the work-unit level.

#### 1.2.6. Cross-Level Effect of TL, EI, WE, and WB to TP and CWB

Contextual model [84] and social cognitive theory (Bandura, 1986) are employed to infer the cross-level relationships. Because organizational-level and individual-level prototypes should be influenced by environmental variables and individual interactions based on the contextual model [84], the cross-level TL should affect TP and CWB by the cross-level EI, WE, and WB at the same time. The work-unit-level variables represent an atmosphere that is shared by every member of the workgroup [85]. In addition, the social cognitive theory also supports the cross-level framework [23] because individual behavior is constituted by the interactive effects between the persona and the surrounding environment. That is to say, an employee who exhibits a high level of TP and CWB is not only affected by WE and CWB (personal perception) at the personal level but also by the cross-level WE and CWB (workgroup atmosphere).

In sum, this survey further posits that the relationships between TL, EI, WE, WB, TP, and CWB at different levels should be functionally similar, and this assumption is supported by previous research [86].

**Hypothesis** **6.** *Work-unit-level TL will affect work-unit-level EI.*

**Hypothesis** **7.** *Work-unit-level EI will affect work-unit-level WE.*

**Hypothesis** **8.** *Work-unit-level EI will affect work-unit-level WB.*

**Hypothesis** **9.** *Work-unit-level WE will affect TP.*

**Hypothesis** **10.** *Work-unit-level WB will affect CWB.*

## 2. Materials and Methods

**Figure 1:**
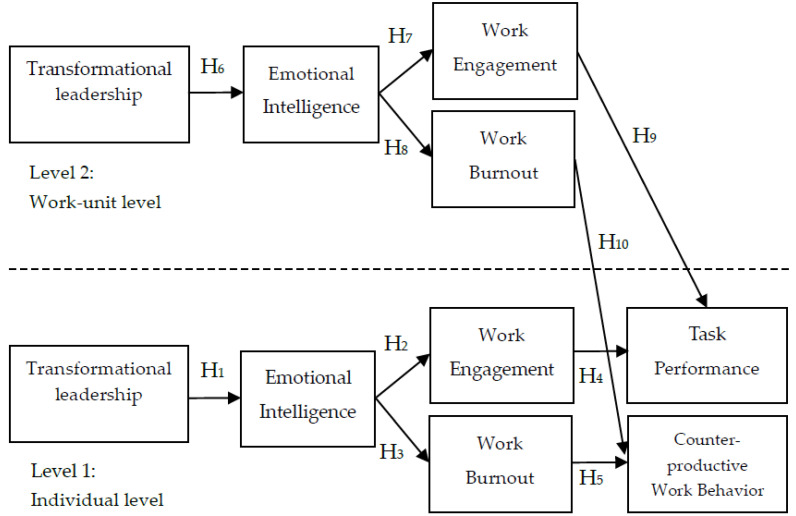
The MGCM of the present research.

This survey constructed a multilevel model that TL results in EL. EI also leads to WE and WB development, which consequently leads to CWB and TP (see Figure 1).

### 2.1. Measures

MLQ Form 5X was adopted to evaluate the ideal infection, inspiration, individual concern, and intelligent excitation of TL [22]. The EI scale developed by Law et al. [87] was adopted to evaluate EI because this scale was developed in Great China setting. The present research employed the scale of Lee and Huang [8] to measure WE because the scale has been confirmed in its reliability and validity in the Greater China context. Singh’s [72] scale was adopted to evaluate TP. WB was evaluated by Ashill and Rod’s [88] scale. Finally, CWB was evaluated by Dalal and his colleague’s [89] scale.

### 2.2. Subjects and Procedures

We approached several agricultural associations in Taiwan and then asked the 80 agribusinesses to provide the email contact of their three livestock feeders with their supervisors to assist our investigation. The sampling list contained 240 livestock feeders. We requested the 240 feeders to assess TL, EI, WE, WB, TP, and CWB. In addition, the data collected from cross-level can significantly improve the bias of the common method [90,91,92].

### 2.3. Validation of Multilevel Data Structure

The reliability and validity of survey data were confirmed by the confirmatory factor analysis, and the results met the standard threshold suggested by Fornell and Larcker [93]. In addition, the model fit (RMR = 0.62, RMSEA = 0.48, CDI = 0.92, GFI = 0.92, NFI = 0.91) is also acceptable. In addition, we adopted the statistics of Shapiro–Wilk to confirm the normal distribution of data, and the statistics are 0.99 (*p* > 0.5).

## 3. Results

### The Results of Analysis

Because the data structure of this study is nested within multiple agribusinesses (consistency within the group but differences between groups), we adopted the multilevel model [36] to analyze the multilevel framework. The 10 Hypotheses are shown as follows, and Table 2 demonstrates the analysis results in the present survey.

**Hypothesis** **1.** *TL will affect EI.*

**Hypothesis** **2.** *EI will affect WE.*

**Hypothesis** **3.** *EI will affect WB.*

**Hypothesis** **4.** *WE will affect TP.*

**Hypothesis** **5.** *WB will affect CWB.*

**Hypothesis** **6.** *Work-unit-level TL will affect work-unit-level EI.*

**Hypothesis** **7.** *Work-unit-level EI will affect work-unit-level WE.*

**Hypothesis** **8.** *Work-unit-level EI will affect work-unit-level WB.*

**Hypothesis** **9.** *Work-unit-level WE will affect TP.*

**Hypothesis** **10.** *Work-unit-level WB will affect CW.*

First, work-unit-level and individual-level TL, respectively, caused work-unit-level EI (*β* = 0.38, *p* *<* 0.01) and individual-level EI (*β* = 0.32, *p* *<* 0.01) development. These results support Hypotheses 1 and 6. That is to say, the work-unit-level and individual-level TL at the first stage affected not only the positive EI atmosphere within the workgroup but also the feeder’s positive EI development.

Second, work-unit-level and individual-level EI, respectively, caused WE at the work-unit level (*β* = 0.36, *p* *<* 0.01) and individual level (*β* = 0.33, *p* *<* 0.01). As a result, Hypotheses 2 and 7 are supported. That is to say, EI at work-unit-level and individual-level caused WE atmosphere within a workgroup and WE.

Third, work-unit-level and individual-level EI, respectively, caused WB at the work-unit level (*β* = −0.35, *p* *<* 0.01) and individual level (*β* = −0.29, *p* *<* 0.01). Hypotheses 3 and 8 are supported. That is to say, EI caused WB atmosphere within a workgroup and WB.

Fourth, WE at work-unit level (*β* = 0.37, *p* *<* 0.01) and individual level (*β* = 0.31, *p* *<* 0.01) caused TP. These results support Hypotheses 4 and 9. That is to say, WE atmosphere within a workgroup and WE caused TP.

Finally, WB at work-unit level (*β* = 0.33, *p* *<* 0.01) and individual level (*β* = 0.28, *p* < 0.01) caused individual-level CWB. These results support Hypotheses 5 and 10. That is to say, WB atmosphere within a workgroup and WB caused the CWB.

## 4. Discussion

### 4.1. The Implications of Academic 

This survey also elucidates the nature of behavioral mechanisms for TP and CWB in that TL can cause EI development to predict such a wide array of behavioral activities. In particular, the WB and CWB should be more serious during the COVID-19 pandemic. In the same vein, EI should be also significantly different during the COVID-19 pandemic. However, TL can still handle these negative behaviors of feeders (WB and CWB) through the intermediary role of EI regardless of whether WB and CWB are more serious. In addition, EI is almost seen as a key antecedent in past studies [94,95,96], but few studies examine how to increase the EI by management method (e.g., leadership). That is to say, the MGCM of the present research makes it possible to cultivate EI, which in turn can deal with a variety of positive and negative behaviors of feeders in agribusinesses. In addition, previous research also supposes that TL may affect EI [59], but there are few studies to empirically investigate this link. 

Finally, the present research also contributes to Kahn’s (1990) [27] theory of WE by considering EI as its antecedent. That is to say, the present research proposes the EI to be one key antecedent of WE rather than the availability, meaningfulness, and safety that Kahn (1990) [27] proposed. In addition, the empirical results that EI is also a key antecedent of WB because EI is a key psychological resource to deal with WB symptoms have been addressed by only a few studies.

### 4.2. The Implications of Practice 

First, in the great explosion of COVID-19, the present research delineated how TL could increase the feeders’ WE and TP development and reduce the feeders’ WB and CWB development in agribusinesses, showing how EI is the key role in handling the two paths. Indeed, although human resource managers must develop intervention strategies for alleviating negative behaviors (e.g., WB and CWB) and enhanced strategies for increasing positive behaviors (e.g., WE and TP), it does not entirely handle this problem. The present research addresses a key path to handle the two concerns at the same time by the TL-EI link. 

Second, during COVID-19, TL can also handle TP and CWB, so these vendors should adopt education training to enhance the TL of supervisors. It makes sense because a transformational leader can adopt ideal infection, inspiration, individual concern, and intelligent excitation to transform employee EI into positive WE, and TP development, and negative WB and CWB development.

Finally, the present research recommends that agribusinesses should invest important resources in improving the EI of employees instead of putting most of the resources in improving multiple motivation and attitudes of feeders because it is more valuable to focus on EI than others.

### 4.3. Further Research and Limitations

This survey poses the key mediating role of EI, but there may be other important variables that can replace the role of EI in different contexts, which leaves further research to explore. Next, although we collected the multilevel data, the causal relationship should be verified by more longitudinal data. Next, the empirical samples in Taiwan may not be generalized to different contexts, so further research should adopt different samples to verify the generalization in the present research. Finally, the number of participants is only 240, so further research should adopt more samples to test the framework in this survey.

## 5. Conclusions 

This survey proposes a multilevel model to argue that the TL can result in TP and CWB through the intermediary effects of EI, WE, and WB. The multilevel model has important contributions to TL, EI, WE, WB, TP, and CWB and can guide agribusinesses to implement sustainable employee career development. Indeed, past studies have rarely adopted this perspective to examine how to deal with negative behaviors and enhance positive behaviors of feeders at the same time. Therefore, the present study proposes that TL can fill the gap and establish a new milestone through the key intermediary role of EI. 

## Figures and Tables

**Table 1:** The acronyms of variables.

Variables	Acronyms
Transformational Leadership	TL
Emotional Intelligence	EI
Work Engagement	WE
Work Burnout	WB
Task Performance	TP
Counterproductive Work Behavior	CWB

**Table 2:** The Results of Analysis.

Hypothesis	Path	Coefficient
H_1_	Individual-level TL→Individual-level EI	0.32 **
H_2_	Individual-level EI→Individual-level WE	0.33 **
H_3_	Individual-level EI→Individual-level WB	−0.29 **
H_4_	Individual-level WE→Individual-level TP	0.31 **
H_5_	Individual-level WB→Individual-level CWB	0.28 **
H_6_	Work-unit-level TL→Work-unit-level EI	0.38 **
H_7_	Work-unit-level EI→Work-unit-level WE	0.36 **
H_8_	Work-unit-level EI→Work-unit-level WB	−0.35 **
H_9_	Work-unit-level WE→Individual-level TP	0.37 **
H_10_	Work-unit-level WB→Individual-level CWB	0.33 **

Notes: TL, transformational leadership; EI, emotional intelligence; WE, work engagement; WB, work burnout; TP, task performance; CWB, counter-productive work behaviors. **: *p* < 0.01.

## Data Availability

Not applicable.

## References

[1] Food and Agriculture Organization of the United Nations (FAO) World Food and Agriculture—Statistical Yearbook 2020. http://www.fao.org/3/cb1329en/online/cb1329en.html.

[2] Narayan E., Barreto M., Hantzopoulou G.-C., Tilbrook A. (2021). A Retrospective Literature Evaluation of the Integration of Stress Physiology Indices, Animal Welfare and Climate Change Assessment of Livestock. Animals.

[3] Hashem N.M., Hassanein E.M., Hocquette J.-F., Gonzalez-Bulnes A., Ahmed F.A., Attia Y.A., Asiry K.A. (2021). Agro-Livestock Farming System Sustainability during the COVID-19 Era: A Cross-Sectional Study on the Role of Information and Communication Technologies. Sustainability.

[4] China Post (2021). Taiwan Unemployment Rate Hits 11-Year High Amid Level 3 Restrictions. https://chinapost.nownews.com/20210723-2663664.

[5] Brozek W., Falkenberg C. (2021). Industrial Animal Farming and Zoonotic Risk: COVID-19 as a Gateway to Sustainable Change? A Scoping Study. Sustainability.

[6] Subedi S., Koirala S., Chai L. (2021). COVID-19 in Farm Animals: Host Susceptibility and Prevention Strategies. Animals.

[7] Zappulli V., Ferro S., Bonsembiante F., Brocca G., Calore A., Cavicchioli L., Centelleghe C., Corazzola G., De Vreese S., Gelain M.E. (2020). Pathology of Coronavirus Infections: A Review of Lesions in Animals in the One-Health Perspective. Animals.

[8] Lee C.-J., Huang S.Y.B. (2020). Double-edged effects of ethical leadership in the development of Greater China salespeople’s emotional exhaustion and long-term customer relationships. Chin. Manag. Stud..

[9] Chang T.-W., Hung C.-Z. (2021). How to Shape the Employees’ Organization Sustainable Green Knowledge Sharing: Cross-Level Effect of Green Organizational Identity Effect on Green Management Behavior and Performance of Members. Sustainability.

[10] Pérez-Sánchez M.d.l.Á., Tian Z., Barrientos-Báez A., Gómez-Galán J., Li H. (2021). Blockchain Technology for Winning Consumer Loyalty: Social Norm Analysis Using Structural Equation Modeling. Mathematics.

[11] Zhu J., Tang W., Wang H., Chen Y. (2021). The Influence of Green Human Resource Management on Employee Green Behavior—A Study on the Mediating Effect of Environmental Belief and Green Organizational Identity. Sustainability.

[12] Du Y., Liu H. (2020). Analysis of the Influence of Psychological Contract on Employee Safety Behaviors against COVID-19. Int. J. Environ. Res. Public Health.

[13] Jia Z., Wen X., Lin X., Lin Y., Li X., Li G., Yuan Z. (2021). Working Hours, Job Burnout, and Subjective Well-Being of Hospital Administrators: An Empirical Study Based on China’s Tertiary Public Hospitals. Int. J. Environ. Res. Public Health.

[14] Liu F., Chen H., Xu J., Wen Y., Fang T. (2021). Exploring the Relationships between Resilience and Turnover Intention in Chinese High School Teachers: Considering the Moderating Role of Job Burnout. Int. J. Environ. Res. Public Health.

[15] Kai Liao Y., Wu W.-Y., Dao T.C., Ngoc Luu T.-M. (2021). The Influence of Emotional Intelligence and Cultural Adaptability on Cross-Cultural Adjustment and Performance with the Mediating Effect of Cross-Cultural Competence: A Study of Expatriates in Taiwan. Sustainability.

[16] Quintana-Orts C., Merida-Lopez S., Rey L., Extremera N. (2021). A Closer Look at the Emotional Intelligence Construct: How Do Emotional Intelligence Facets Relate to Life Satisfaction in Students Involved in Bullying and Cyberbullying?. Eur. J. Investig. Health Psychol. Educ..

[17] Salovey P., Mayer J.D. (1990). Emotional intelligence. Imagin. Cogn. Pers..

[18] Aziz F., Md Rami A.A., Zaremohzzabieh Z., Ahrari S. (2021). Effects of Emotions and Ethics on Pro-Environmental Behavior of University Employees: A Model Based on the Theory of Planned Behavior. Sustainability.

[19] García-Martínez I., Pérez-Navío E., Pérez-Ferra M., Qauijano-López R. (2021). Relationship between Emotional Intelligence, Educational Achievement and Academic Stress of Pre-Service Teachers. Behav. Sci..

[20] Montenegro A., Dobrota M., Todorovic M., Slavinski T., Obradovic V. (2021). Impact of Construction Project Managers’ Emotional Intelligence on Project Success. Sustainability.

[21] Brown F.W., Moshavi D. (2005). Transformational leadership and emotional intelligence: A potential pathway for an increased understanding of interpersonal influence. J. Organ. Behav..

[22] Bass B.M., Avolio B.J. (1995). MLQ Multifactor Leadership Questionnaire for Research.

[23] Bandura A. (1986). Social Foundations of Thought and Action: A Social Cognitive Theory.

[24] Mayer J.D., Salovey P., Caruso D.R. (2008). Emotional intelligence: New ability or eclectic traits?. Am. Psychol..

[25] Maslach C., Schaufelli W.B., Leiter M.P. (2001). Job burnout. Annu. Rev. Psychol..

[26] Fox S., Spector P.E., Miles D. (2001). Counterproductive work behavior (CWB) in response to job stressors and organizational justice: Some mediator and moderator tests for autonomy and emotions. J. Vocat. Behav..

[27] Kahn W.A. (1990). Psychological conditions of personal engagement and disengagement at work. Acad. Manag. J..

[28] Liu C., Cao J., Zhang P., Wu G. (2020). Investigating the Relationship between Work-To-Family Conflict, Job Burnout, Job Outcomes, and Affective Commitment in the Construction Industry. Int. J. Environ. Res. Public Health.

[29] Ha D.-J., Park J.-H., Jung S.-E., Lee B., Kim M.-S., Sim K.-L., Choi Y.-H., Kwon C.-Y. (2021). The Experience of Emotional Labor and Its Related Factors among Nurses in General Hospital Settings in Republic of Korea: A Systematic Review and Meta-Analysis. Sustainability.

[30] Tabak F., Tziner A., Shkoler O., Rabenu E. (2021). The Complexity of Heavy Work Investment (HWI): A Conceptual Integration and Review of Antecedents, Dimensions, and Outcomes. Sustainability.

[31] Bakker A.B., Demerouti E., Schaufeli W.B. (2005). The crossover of burnout and work engagement among working couples. Hum. Relat..

[32] Park J.H., Chang Y.K., Kim S. (2021). Are Your Vitals OK? Revitalizing Vitality of Nurses through Relational Caring for Patients. Healthcare.

[33] Kim J., Lee S., Byun G. (2020). Building a Thriving Organization: The Antecedents of Job Engagement and Their Impact on Voice Behavior. Sustainability.

[34] Mazzetti G., Guglielmi D., Schaufeli W.B. (2020). Same Involvement, Different Reasons: How Personality Factors and Organizations Contribute to Heavy Work Investment. Int. J. Environ. Res. Public Health.

[35] Vila-Vazquez G., Castro-Casal C., Alvarez-Perez D., Del Rio-Araujo L. (2018). Promoting the Sustainability of Organizations: Contribution of Transformational Leadership to Job Engagement. Sustainability.

[36] Raudenbush S.W., Bryk A.S. (2002). Hierarchical Linear Models: Applications and Data Analysis Methods.

[37] Huang S.Y.B., Chen K.-H., Lee Y.-S. (2021). How to Promote Medium-Sized Farms to Adopt Environmental Strategy to Achieve Sustainable Production during the COVID-19 Pandemic?. Agriculture.

[38] Huang S.Y.B., Lee S.-C., Lee Y.-S. (2021). Constructing an Adoption Model of Proactive Environmental Strategy: A Novel Quantitative Method of the Multi-Level Growth Curve Model. Mathematics.

[39] Kim D.G., Lee C.W. (2021). Exploring the Roles of Self-Efficacy and Technical Support in the Relationship between Techno-Stress and Counter-Productivity. Sustainability.

[40] Yu Y., Cheng H. (2021). Environmental Taxes and Innovation in Chinese Textile Enterprises: Influence of Mediating Effects and Heterogeneous Factors. Sustainability.

[41] Szostek D. (2019). The Impact of the Quality of Interpersonal Relationships between Employees on Counterproductive Work Behavior: A Study of Employees in Poland. Sustainability.

[42] Carvalho V.S., Santos A., Ribeiro M.T., Chambel M.J. (2021). Please, Do Not Interrupt Me: Work–Family Balance and Segmentation Behavior as Mediators of Boundary Violations and Teleworkers’ Burnout and Flourishing. Sustainability.

[43] Çelik A.A., Kılıç M., Altindağ E., Öngel V., Günsel A. (2021). Does the Reflection of Foci of Commitment in Job Performance Weaken as Generations Get Younger? A Comparison between Gen X and Gen Y Employees. Sustainability.

[44] Miao Q., Zhou J. (2020). Corporate Hypocrisy and Counterproductive Work Behavior: A Moderated Mediation Model of Organizational Identification and Perceived Importance of CSR. Sustainability.

[45] Rasool S.F., Wang M., Tang M., Saeed A., Iqbal J. (2021). How Toxic Workplace Environment Effects the Employee Engagement: The Mediating Role of Organizational Support and Employee Wellbeing. Int. J. Environ. Res. Public Health.

[46] Sanchez-Pujalte L., Mateu D.N., Etchezahar E., Gomez Yepes T. (2021). Teachers’ Burnout during COVID-19 Pandemic in Spain: Trait Emotional Intelligence and Socioemotional Competencies. Sustainability.

[47] Antonakis J., Ashkanasy N.M., Dasborough M.T. (2009). Does leadership need emotional intelligence?. Leadersh. Q..

[48] You M., Laborde S., Zammit N., Iskra M., Borges U., Dosseville F., Vaughan R.S. (2021). Emotional Intelligence Training: Influence of a Brief Slow-Paced Breathing Exercise on Psychophysiological Variables Linked to Emotion Regulation. Int. J. Environ. Res. Public Health.

[49] Huang S.Y.B., Ting C.-W., Li M.-W. (2021). The Effects of Green Transformational Leadership on Adoption of Environmentally Proactive Strategies: The Mediating Role of Green Engagement. Sustainability.

[50] Huang S.Y.B., Fei Y.-M., Lee Y.-S. (2021). Predicting Job Burnout and Its Antecedents: Evidence from Financial Information Technology Firms. Sustainability.

[51] Huang S.Y.B., Li M.-W., Chang T.-W. (2021). Transformational Leadership, Ethical Leadership, and Participative Leadership in Predicting Counterproductive Work Behaviors: Evidence From Financial Technology Firms. Front. Psychol..

[52] Clements H., Valentin S., Jenkins N., Rankin J., Gee N.R., Snellgrove D., Sloman K.A. (2021). Companion Animal Type and Level of Engagement Matter: A Mixed-Methods Study Examining Links between Companion Animal Guardianship, Loneliness and Well-Being during the COVID-19 Pandemic. Animals.

[53] Janssens M., Janssens E., Eshuis J., Lataster J., Simons M., Reijnders J., Jacobs N. (2021). Companion Animals as Buffer against the Impact of Stress on Affect: An Experience Sampling Study. Animals.

[54] Kogan L.R., Currin-McCulloch J., Bussolari C., Packman W., Erdman P. (2021). The Psychosocial Influence of Companion Animals on Positive and Negative Affect during the COVID-19 Pandemic. Animals.

[55] Changar M., Atan T. (2021). The Role of Transformational and Transactional Leadership Approaches on Environmental and Ethical Aspects of CSR. Sustainability.

[56] Gurmani J.K., Khan N.U., Khalique M., Yasir M., Obaid A., Sabri N.A.A. (2021). Do Environmental Transformational Leadership Predicts Organizational Citizenship Behavior towards Environment in Hospitality Industry: Using Structural Equation Modelling Approach. Sustainability.

[57] Zaman U., Nawaz S., Nadeem R.D. (2020). Navigating Innovation Success through Projects. Role of CEO Transformational Leadership, Project Management Best Practices, and Project Management Technology Quotient. J. Open Innov. Technol. Mark. Complex..

[58] Peter P.C., Brinberg D. (2012). Learning Emotional Intelligence: An Exploratory Study in the Domain of Health. J. Appl. Soc. Psychol.

[59] Coetzee C., Schaap P. (2005). The relationship between leadership behaviour, outcomes of leadership and emotional intelligence. SA J. Ind. Psychol..

[60] Hobfoll S.E., Johnson R.J., Ennis N.E., Jackson A.P. (2003). Resource loss, resource gain, and emotional outcomes among inner-city women. J. Personal. Soc. Psychol..

[61] Fredrickson B. (2004). The broaden-and-build theory of positive emotions. Philos. Trans. R. Soc. London Biol. Sci..

[62] Tugade M., Fredrickson B. (2004). Resilient individuals use emotions to bounce back from negative emotional experiences. J. Personal. Soc. Psychol..

[63] Giordano B. (1997). Resilience: A survival tool for the nineties. AORN J..

[64] Meijman T.F., Mulder G., Drenth P.J.D., Hove H.T. (1998). Psychological aspects of workload in Handbook of work and organizational psychology. Work Psychology.

[65] Tims M., Bakker A.B., Xanthopoulou D. (2011). Do transformational leaders enhance their followers’ daily work engagement?. Leadersh. Q..

[66] Choi S., Kang Y., Yeo K. (2021). Effect of a Protestant Work Ethic on Burnout: Mediating Effect of Emotional Dissonance and Moderated Mediating Effect of Negative Emotion Regulation. Sustainability.

[67] Fiorilli C., Buonomo I., Romano L., Passiatore Y., Iezzi D.F., Santoro P.E., Benevene P., Pepe A. (2020). Teacher Confidence in Professional Training: The Predictive Roles of Engagement and Burnout. Sustainability.

[68] Geng L.N., Li S.S., Zhou W.J. (2011). The Relationships among Emotional Exhaustion, Emotional Intelligence, and Occupational Identity of Social Workers in China. Soc. Behav. Personal..

[69] Tsaousis I., Nikolaou I. (2005). Exploring the relationship of emotional intelligence with physical and psychological health functioning. Stress Health.

[70] Hobfoll S.E., Freedy J. (1993). Professional Burnout: Recent Developments in Theory and Research.

[71] Lee R.T., Ashforth B.E. (1996). A Meta-Physic Examination of the Correlates of Three Dimensions of Job Burnout. J. Appl. Psychol..

[72] Singh S.K. (2019). Territoriality, task performance, and workplace deviance: Empirical evidence on role of knowledge hiding. J. Bus. Res..

[73] Schaufeli W.B., Bakker A.B. (2004). Job demands, job resources, and their relationship with burnout and engagement: A multi-sample study. J. Organ. Behav..

[74] Banks G.C., Whelpley C.E., Oh I.-S., Shin K. (2012). (How) are emotionally exhausted employees harmful?. Int. J. Stress Manag..

[75] Krischer M.M., Penney L.M., Hunter E.M. (2010). Can counterproductive work behaviors be productive? CWB as emotion-focused coping. J. Occup. Health Psychol..

[76] Hobfoll S.E. (1989). Conservation of resources: A new attempt at conceptualizing stress. Am. Psychol..

[77] Rahmadani V.G., Schaufeli W.B., Stouten J., Zhang Z., Zulkarnain Z. (2020). Engaging Leadership and Its Implication for Work Engagement and Job Outcomes at the Individual and Team Level: A Multi-Level Longitudinal Study. Int. J. Environ. Res. Public Health.

[78] Rezvani A., Khosravi P., Ashkanasy N.M. (2018). Examining the interdependencies among emotional intelligence, trust, and performance in infrastructure projects: A multilevel study. Int. J. Proj. Manag..

[79] Xu W., Pan Z., Li Z., Lu S., Zhang L. (2020). Job Burnout Among Primary Healthcare Workers in Rural China: A Multilevel Analysis. Int. J. Environ. Res. Public Health.

[80] Zhang Y., Zheng J., Darko A. (2018). How Does Transformational Leadership Promote Innovation in Construction? The Mediating Role of Innovation Climate and the Multilevel Moderation Role of Project Requirements. Sustainability.

[81] Kozlowski S.W.J., Klein K.J., Klein K.J., Kozlowski S.W.J. (2000). A multilevel approach to theory and research in organizations: Contextual, temporal, and emergent processes. Multilevel Theory, Research, and Methods in Organizations: Foundations, Extensions, and New Directions.

[82] Cappelli P., Sherer P.D., Cummings L.L., Staw B.M. (1991). The missing role of context in OB: The need for a meso-level approach. Research in Organizational Behavior.

[83] Louis M.R., Posner B.Z., Powell G.N. (1983). The availability and helpfulness of socialization practices. Pers. Psychol..

[84] Firebaugh G., Roberts K.H., Burstein L. (1980). Groups as contexts and frog ponds. Issues in Aggregation.

[85] Hackman J.R., Dunnette M.D., Hough L.M. (1992). Group influences on individuals in organizations. Handbook of Industrial Organizational Psychology.

[86] Chen G., Kanfer R., Staw B.M. (2006). Toward a systems theory of motivated behavior in work team. Research in Organizational Behavior.

[87] Law K.S., Song L.J., Wong C.S. (2004). The Construct and Criterion Validity of Emotional Intelligence and Its Potential Utility for Management Studies. J. Appl. Psychol..

[88] Ashill N.J., Rod M. (2011). Burnout processes in non-clinical health service encounters. J. Bus. Res..

[89] Dalal R.S., Lam H., Weiss H.M., Welch E.R., Hulin C.L. (2009). A Within-Person Approach to Work Behavior and Performance: Concurrent and Lagged Citizenship-Counterproductivity Associations, and Dynamic Relationships with Affect and Overall Job Performance. Acad. Manag. J..

[90] Huang S.Y.B., Ting C.-W., Fei Y.-M. (2021). A Multilevel Model of Environmentally Specific Social Identity in Predicting Environmental Strategies: Evidence from Technology Manufacturing Businesses. Sustainability.

[91] Huang S.Y.B., Li M.-W., Lee Y.-S. (2021). Why Do Medium-Sized Technology Farms Adopt Environmental Innovation? The Mediating Role of Pro-Environmental Behaviors. Horticulturae.

[92] Podsakoff P.M., MacKenzie S.B., Lee J., Podsakoff N.P. (2003). Common method biases in behavioral research: A critical review of the literature and recommended remedies. J. Appl. Psychol..

[93] Fornell C., Lacker D.F. (1981). Evaluating structural equation models with unobservable variables and measurement error. J. Mark. Res..

[94] Blazquez Puerta C.D., Bermudez Gonzalez G., Soler Garcia I.P. (2021). Executives’ Knowledge Management and Emotional Intelligence Role: Dynamizing Factor towards Open Innovation. J. Open Innov. Technol. Mark. Complex..

[95] Llinares-Insa L.I., Casino-Garcia A.M., Garcia-Perez J. (2020). Subjective Well-Being, Emotional Intelligence, and Mood of Parents: A Model of Relationships. Impact of Giftedness. Sustainability.

[96] Nateri R., Robazza C., Tolvanen A., Bortoli L., Hatzigeorgiadis A., Ruiz M.C. (2020). Emotional Intelligence and Psychobiosocial States: Mediating Effects of Intra-Team Communication and Role Ambiguity. Sustainability.

